# Supplementary material for: mRNA Vaccines Enhance Neutralizing Immunity against SARS-CoV-2 Variants in Convalescent and ChAdOx1-Primed Subjects
Source: Vaccines (Basel). 2021 Aug 18;9(8):918. doi: 10.3390/vaccines9080918 (PMC8402682; doi:10.3390/vaccines9080918)
Supplement: Supplementary file 1 [file vaccines-09-00918-s001.zip › vaccines-1314960-supplementary.pdf]

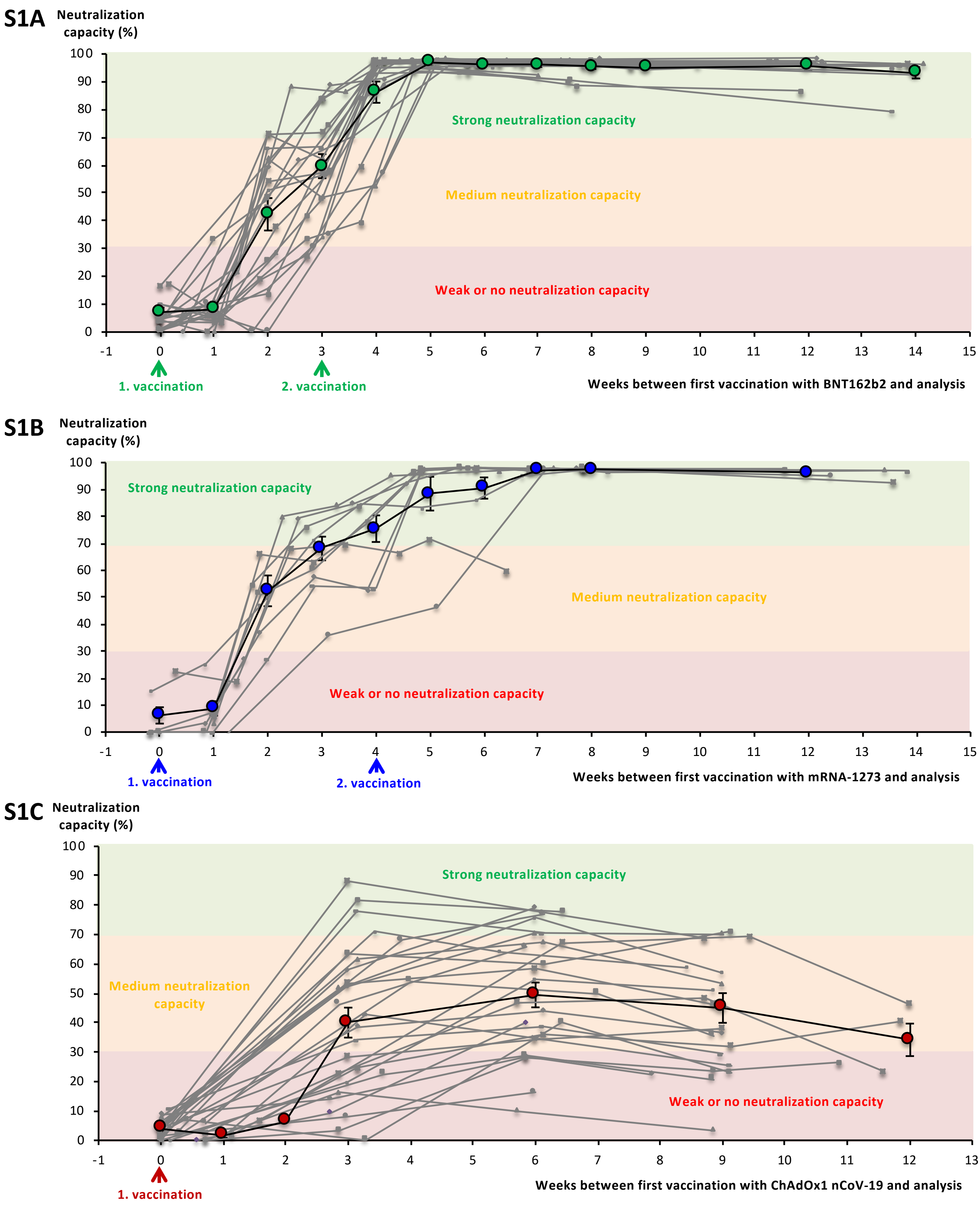

Supplementary Figure 1. Individual courses of neutralization titers after vaccination against SARS-CoV-2. Serum samples from (A) 21 fully vaccinated BNT162b2 vaccinees, (B) 10 fully vaccinated mRNA-1273 vaccinees and (C) 29 ChAdOx1-nCoV-19 vaccinees were collected at different time points after first vaccination as indicated and tested for neutralization of ACE2-RBD interaction. Plotted are individual (grey lines) and average (colored circles) results. Error bars indicate SEM. Abbreviations: ACE2: angiotensin-converting enzyme 2, RBD: receptor-binding domain.

S2A

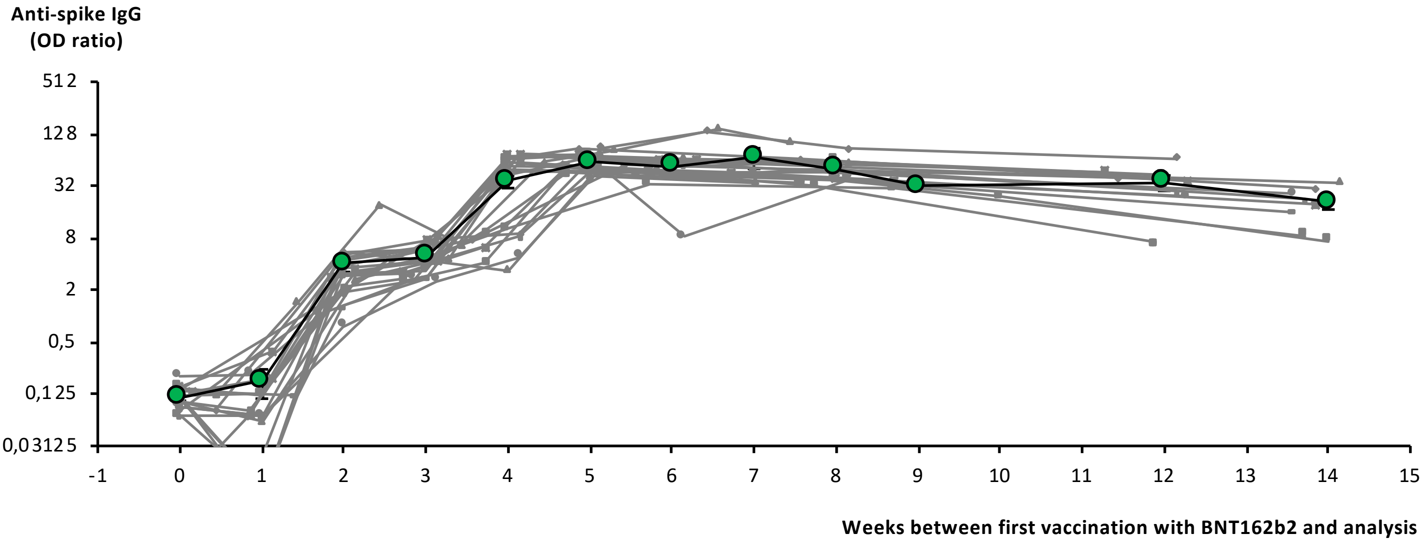

S2B

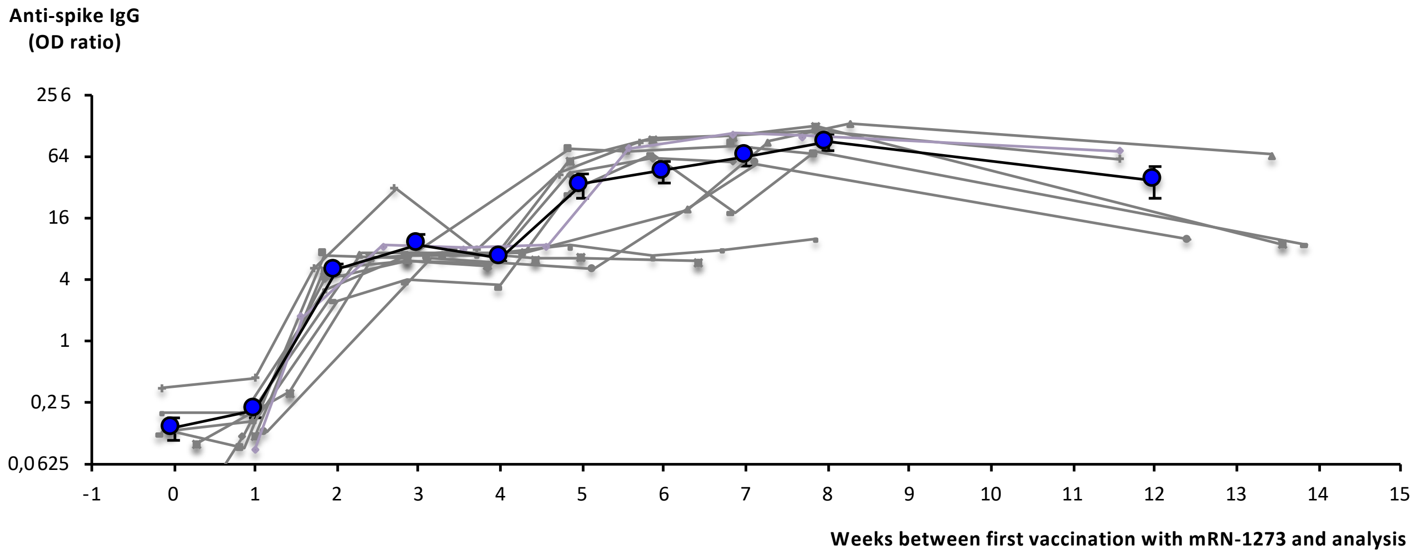

S2C

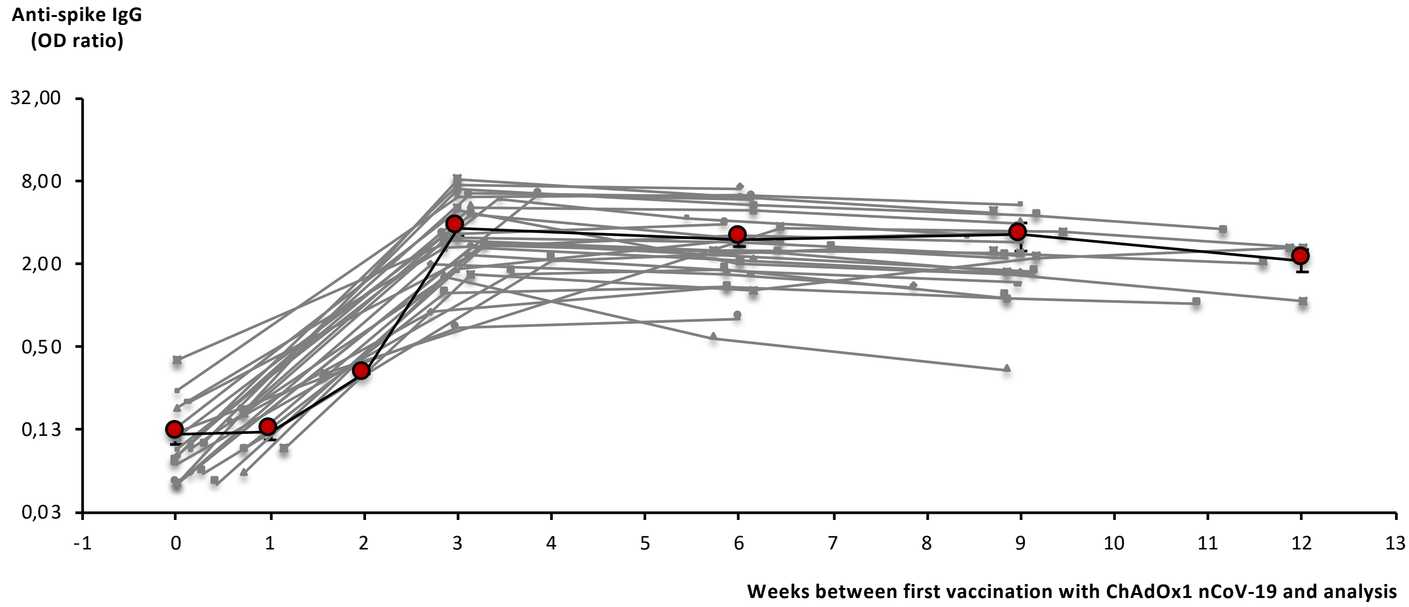

Supplementary Figure 2. Individual courses of anti-spike IgG titers after vaccination against SARS-CoV-2. Serum samples from (A) 21 fully vaccinated BNT162b2 vaccinees, (B) 10 fully vaccinated mRNA-1273 vaccinees and (C) 29 ChAdOx1 nCoV-19 vaccinees were collected at different time points after first vaccination as indicated and tested for anti-spike IgG titers. Plotted are individual (grey lines) and average (colored circles) results. Error bars indicate SEM. Abbreviations: OD: optical density.

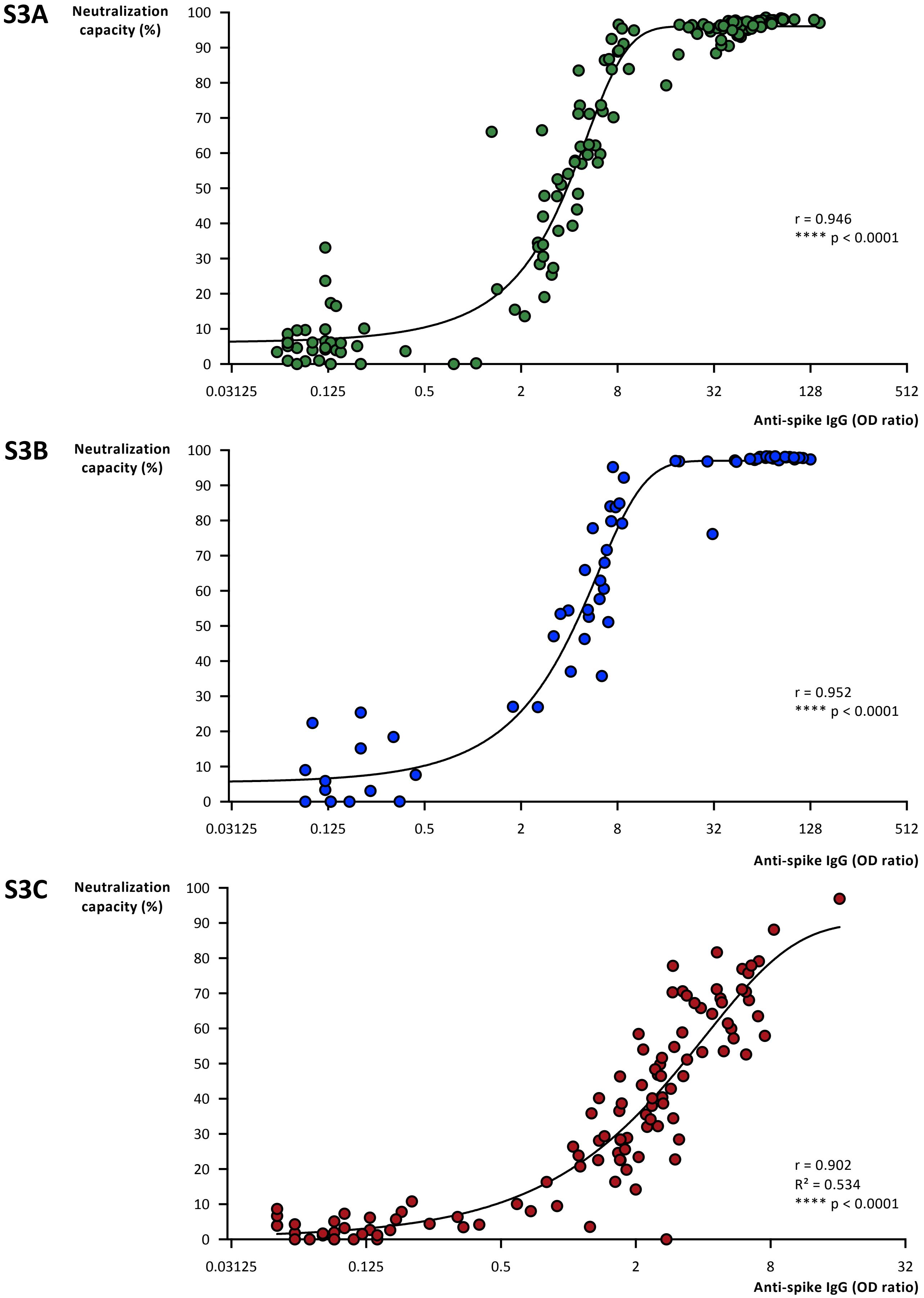

Supplementary Figure 3. Correlation between anti-spike IgG and neutralization titers.
172 serum samples from 21 BNT162b2 (A), 66 serum samples from 10 mRNA1273 (B) and 110 serum samples from 29 ChAdOx1 nCoV-19 (C) vaccinees were collected at different time points after first vaccination. Subsequently samples were tested using a neutralization test based on the inhibition of SARS-CoV-2 RBD interaction with the ACE2 protein, and an ELISA for anti-spike IgG. Dot plots show correlation between both test results. **** p values < 0.0001 indicate highly significant Spearman correlation. Abbreviations: RBD: receptor binding domain; ACE2: angiotensin-converting enzyme 2.

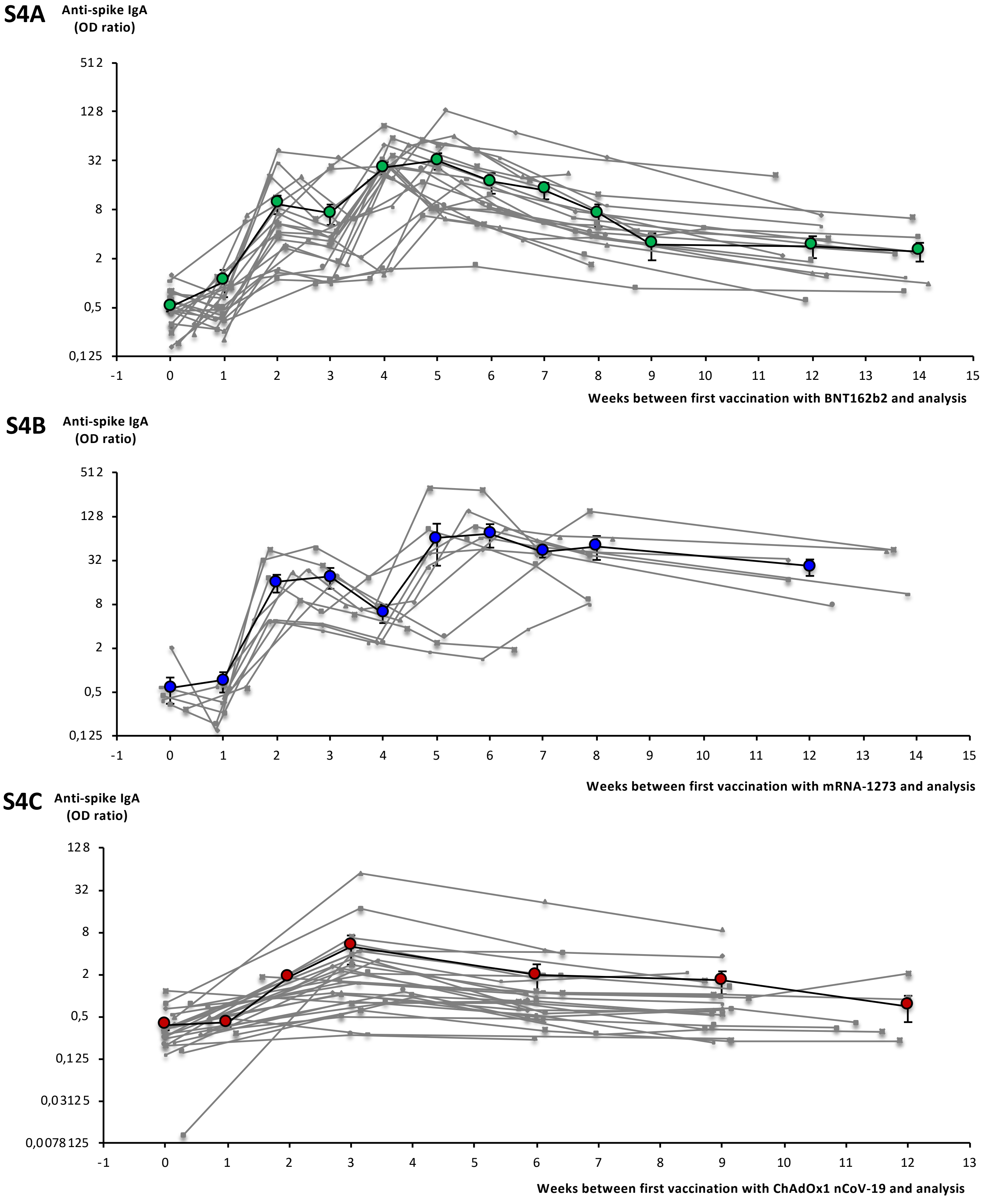

Supplementary Figure 4. Individual courses of anti-spike IgG titers after vaccination against SARS-CoV-2. Serum samples from (A) 21 fully vaccinated BNT162b2 vaccinees, (B) 10 fully vaccinated mRNA-1273 vaccinees and (C) 29 ChAdOx1 nCoV-19 vaccinees were collected at different time points after first vaccination as indicated and tested for anti-spike IgA titers. Plotted are individual (grey lines) and average (colored circles) results. Error bars indicate SEM. Abbreviations: OD: optical density.

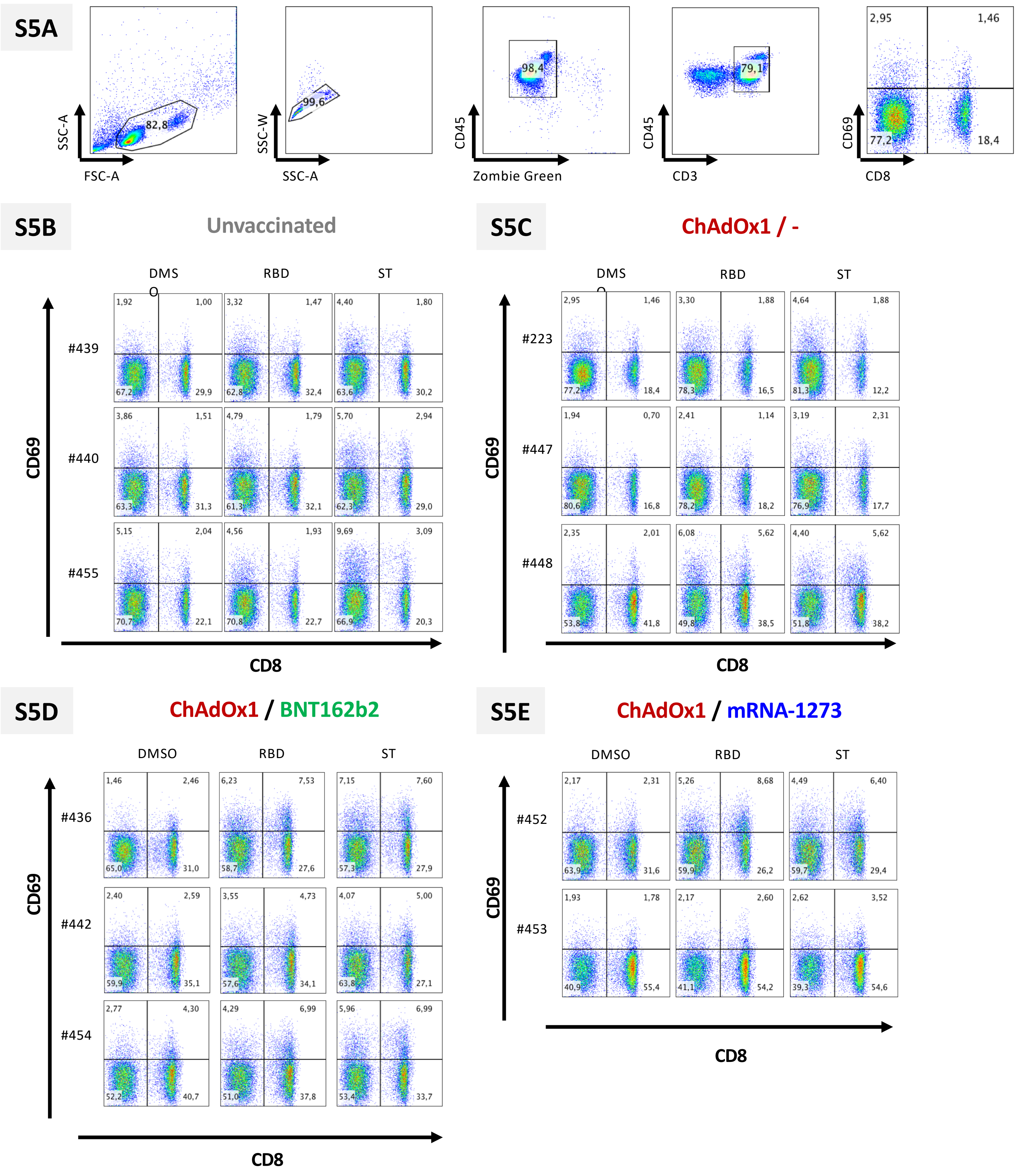

Supplementary Figure 5. Differential SARS-CoV-2-spike-specific T cell response after vaccination.
Freshly isolated PBMC from (B) three unvaccinated control individuals, (C) three individuals vaccinated with ChAdOx1-nCoV-19 alone, (D three individuals vaccinated with ChAdOx1-nCoV-19 and BNT162b2), and (E) two individuals vaccinated with ChAdOx1-nCoV-19 and mRNA-1273 were incubated for 48 hours in the presence of either a peptide mix representing the receptor-binding domain (RBD) of the SARS-CoV-2-spike protein, a peptide pool representing a broad variety of recall antigens (ST), or the solvent DMSO as control. Then, cells were harvested, stained and analyzed using multi-parameter FACS analysis. Panel (A) shows the gating strategy, panels (B-E) show individual results in the four groups indicated above. For clarity reasons, CD4 staining is not shown, although it was performed. Abbreviations: DMSO: Dimethyl Sulfoxide, RBD: RBD: receptor-binding domain, ST: Recall antigen stimulation cocktail.
